# P53 functional abnormality in mesenchymal stem cells promotes osteosarcoma development

**DOI:** 10.1038/cddis.2015.367

**Published:** 2016-01-21

**Authors:** T Velletri, N Xie, Y Wang, Y Huang, Q Yang, X Chen, Q Chen, P Shou, Y Gan, G Cao, G Melino, Y Shi

**Affiliations:** 1 Key Laboratory of Stem Cell Biology, Institute of Health Sciences, Shanghai Institutes for Biological Sciences, Chinese Academy of Sciences/Shanghai Jiao Tong University, School of Medicine, 320 Yueyang Road, Shanghai 200031, China; 2 Biochemistry Laboratory IDI-IRCC, Department of Experimental Medicine and Surgery, University of Rome Torvergata, Rome 00133, Italy; 3 Medical Research Council, Toxicology Unit, Leicester University, Leicester LE1 9HN, UK; 4 Soochow Institutes for Translational Medicine, Soochow University, Suzhou, China

## Abstract

It has been shown that p53 has a critical role in the differentiation and functionality of various multipotent progenitor cells. P53 mutations can lead to genome instability and subsequent functional alterations and aberrant transformation of mesenchymal stem cells (MSCs). The significance of p53 in safeguarding our body from developing osteosarcoma (OS) is well recognized. During bone remodeling, p53 has a key role in negatively regulating key factors orchestrating the early stages of osteogenic differentiation of MSCs. Interestingly, changes in the p53 status can compromise bone homeostasis and affect the tumor microenvironment. This review aims to provide a unique opportunity to study the p53 function in MSCs and OS. In the context of loss of function of p53, we provide a model for two sources of OS: MSCs as progenitor cells of osteoblasts and bone tumor microenvironment components. Standing at the bone remodeling point of view, in this review we will first explain the determinant function of p53 in OS development. We will then summarize the role of p53 in monitoring MSC fidelity and in regulating MSC differentiation programs during osteogenesis. Finally, we will discuss the importance of loss of p53 function in tissue microenvironment. We expect that the information provided herein could lead to better understanding and treatment of OS.

## Facts

- P53 is a guardian of cell differentiation.
- P53 regulates genomic stability, growth, proliferation, and immunoproperties of mesenchymal stem cells (MSCs).
- P53 is a negative regulator of osteogenic differentiation of MSCs.
- Loss of function of p53 in MSCs compromises their osteogenic differentiation and affects the properties of bone tumor microenvironment (BME) components, therefore it dictates the conditions for osteosarcoma (OS) development.

## Open Questions

- To identify *in vivo* and *in vitro* key molecules involved in the process of bone remodeling, in the context of loss of function of p53.
- Are there any molecules produced by p53-null MSCs that could affect osteoclast properties and compromise bone homeostasis?
- How do they relate to the diagnosis and prognosis of OS?

TP53 belongs to the so-called ‘p53 gene family' of transcription factors, which includes also the proteins p63, p73, and p53 itself.^1, 2, 3^ Having been discovered since 1979, p53 is the most studied member of the family with over 60 000 papers so far published. This large mass of scientific data evidentiate a huge complexity for p53 functional program, ranging from the regulation of metabolism^4, 5, 6^ and mitochondria/oxygen radicals^7, 8^ to the deeply analyzed DNA damage repair system,^9, 10, 11, 12, 13, 14^ autophagy,^15, 16^ and, last but not the least, its role in cell stem maintenance and lineage determination.^17, 18^ Despite all these investigations, efforts, and advances in knowledge, many crucial intriguing points still remain unanswered to fully understand the physiological and pathological role of p53. These wide range of effects raise from several angles, including, for example, its regulation at the transcriptional level, at the level of micro-RNA,^19, 20, 21, 22^ and splicing isoforms^23, 24^ to its translational regulation and its stability/degradation at the protein level.^25, 26, 27, 28, 29^ In parallel to so much effort in understanding the function of p53, significant efforts are also underway on its potential clinical exploitation.^30, 31, 32, 33, 34, 35, 36, 37^ Although being identified after ~20 years, already now, p63 and p73 show a similar complexity, and also the ability to interact with p53 at the structural and functional level,^34, 38, 39, 40, 41, 42, 43, 44, 45, 46, 47, 48, 49^ where the p63 function is highly relevant in skin formation and homeostasis,^50, 51^ as well as in cancer^46, 52, 53^ and stem cell regulation.^54, 55, 56, 57^

## P53 and OS in clinical settings

### P53 and tumor

The p53 family of transcription factors have several members including p53, p63, and p73. Each member of this family expresses unique mRNA variants resulting from alternative splicing, promoters, and transcription initiation sites.^58^ Thus, a single gene can exist in multiple isoforms with distinct biological functions.^59, 60^ P53 protein, encoded by the *TP53* gene in humans and the *Trp53* gene in mice, is well known for its role as the ‘guardian of the genome' and exerts a pivotal role in maintaining the genetic stability.^61, 62, 63^ It can prevent tumor formation by regulating cell cycle,^64^ apoptosis,^65^ senescence,^66^ and metabolism^67^ by binding to responsive elements on DNA (p53RE).^64, 68^ Abnormal regulation of the p53 family has a critical role in tumorigenesis; indeed, *TP53* mutations have been detected in over 50% of all human cancers.^69, 70^

Silent mutations in the tumor suppressor gene *TP53* and/or the retinoblastoma gene *RB1* have been reported to be the main causes of the development of sporadic OS.^71^ *In vitro* experiments comparing MSCs with malignant OS cells, as well as *in vivo* studies using transgenic mice with targeting p53 and Rb (retinoblastoma gene *RB1*; retinoblastoma protein) silencing in MSCs, have elegantly demonstrated that when p53 alone was deleted, the incidence of OS could reach 60%.^72^ Another function of p53 in suppressing tumor is to act as ‘a guardian of differentiation'.^59^

Notably, p53 guards osteogenic, myogenic, adipogenic, hematopoietic, and neural differentiation of adult stem cells.^73, 74^

### P53 in OS

**Figure 1:**
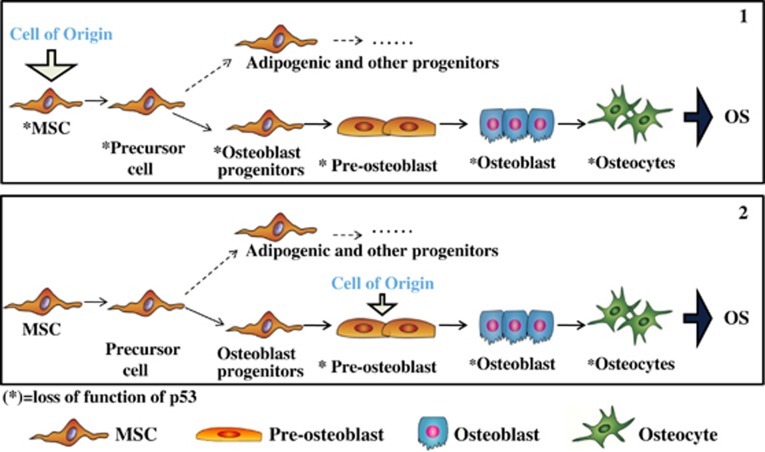
Loss of function of p53 in undifferentiated MSCs and origin of OS. Preosteoblasts and osteoblasts can be considered as cells of origin for OS development (2) as well as undifferentiated MSCs (1). When mesenchymal progenitor cells (1) or preosteoblast cells (2) become aberrant following mutational events of p53 tumor suppressor gene (*p53), they show compromised growth, proliferation and terminal differentiation. The arrow (*) indicates MSCs with loss of function of p53: *MSCs

OS is a bone tumor affecting long bones in childhood and adolescence.^75^ Seven subtypes of OS have been characterized according to histological analysis of the osteoid matrix produced by aberrant osteoblasts: osteoblastic, fibroblastic, chondroblastic, telangiectatic, epithelial, small cell, and giant-rich cell.^76^ The abundant deposition of osteoid matrix and osteoblast-like features of the malignant cells are the dominant characteristics of the osteoblastic phenotype. This subtype manifests the highest incidence representing 75% of screened OS.^76, 77, 78^ Within OS of the osteoblastic subtype, aberrant preosteoblasts and osteoblasts produce their own osteoid mineralized matrix close to the area of growth plate (GP). Although chromosomal abnormalities have a decisive role in the development of OS,^79^ the karyotype is not essential for the subtype classification. OS frequently occurs in human patients with Li-Fraumeni syndrome and with hereditary retinoblastoma. Li-Fraumeni patients carry a germline p53 mutation in one allele compromising the function of p53.^80, 81, 82^ Different studies have reported that preosteoblasts and osteoblasts represent the cells of origin of OS.^78, 83^ Importantly, cellular microenvironment is also decisive in determining the fate of stem cells and in promoting tumor formation.^84^ The osteogenic differentiation of p53-deficient or mutant MSCs can be affected by signals from BME and promote eventually OS. Intrabone inoculation of undifferentiated p53^−/−^ and p53^−^^/^^−^Rb^−/−^ MSCs generated osteoblastic OS and developed metastasis characterized by osteoid areas in the lung, spleen, and heart.^33^ These data suggest that, along with specific bone microenvironment conditions, undifferentiated MSCs with compromised p53 function can represent the cells of origin of OS (Figure 1). The initiation of the tumor could, in part, be affected by a failure of MSCs in maintaining a balance with other differentiation gene programs, such as adipogenesis and chondrogenesis.^78^ Notably, p53^−/−^Rb^−/−^ MSCs reflect the phenotype for the development of sarcoma.^79^ Interestingly, the feature of the osteoblast subtype of OS is tightly associated with the impaired activity of p53 in mouse MSCs and osteoblasts.^85^

## Formation of OS

### Aberrant proliferation of preosteoblasts and osteoblasts

**Figure 2:**
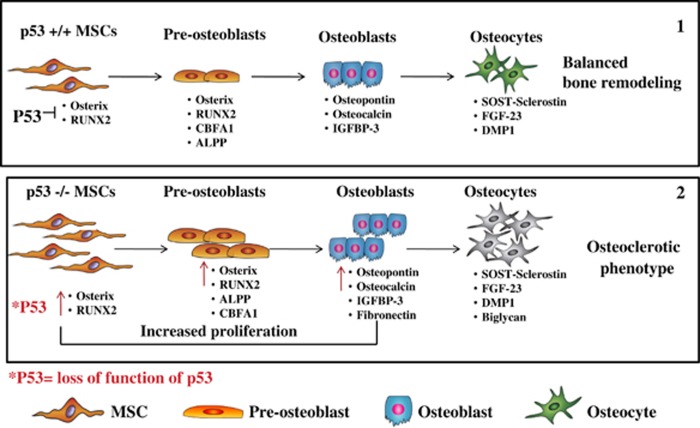
P53-null MSCs show abnormal osteogenesis compared with the wild-type MSCs. In non-aberrant conditions, the expression of Osterix and Runx2 is upregulated during osteogenic differentiation of osteogenic committed cells to promote their differentiation and maturation towards osteoblasts and osteocytes, and to ensure a balanced bone remodeling (1). P53-null MSCs express before the commitment towards upregulated levels of both Osterix and Runx2. This compromises their differentiation towards mature osteoblasts and osteocytes, culminating in impaired bone remodeling and in the osteosclerotic phenotype observed in p53-deficient mice (2)

The bone is an alive and active tissue, crossed by blood vessels that form a complex sinusoidal vascular network. Its basic structure is composed of both trabecular and cortical bone, with trabecules of trabecular bone interspersed in the bone marrow and in direct contact with the bone marrow microenvironment. The surface of the trabecules includes both active and quiescent osteoblasts, which originate the endosteal niche.^86^ During bone remodeling, the osteoblasts, cells of mesenchymal origin,^87^ and the osteoclasts, cells of hematopoietic origin, cooperate and work in proximity of the endosteum niche, respectively, generating and resorbing the bone. Osteoblasts and osteoclasts can communicate, regulate, and activate each other through the secretion of specific key molecules (coupling growth factors) released during bone resorption. Insulin-like growth factors and transforming growth factor-beta (TGF-*β*) are examples of two coupling growth factors secreted during bone degradation, which have been proven to stimulate the osteoblast activity.^88^ However, MSCs and osteoblasts can also secrete molecules that can influence the osteoclast activity, and affect bone remodeling in the same cases.^89^ Indeed, throughout the lifetime bone remodeling is kept at a constant rate to balance bone formation and bone degradation, and to guarantee bone homeostasis. However, along with aging, this exquisite equilibrium is subjected to alterations mainly as a consequence of hormonal dysfunctions. Indeed, steroid hormone deficit enhances the resorbing activity of osteoclasts, which could terminate in an osteoporotic condition.^90^ Conversely, an increase in bone mass and bone density is representative of osteosclerosis and osteopetrosis conditions. Higher intake of bone in osteosclerosis is exclusively promoted by osteoblasts, whereas osteopetrosis is due to aberrant activity of osteoclast-mediated bone resorption.^91^ Notably, during the process of endochondral bone formation, which occurs until adolescence, the mesenchymal progenitor cells first differentiate into chondrocytes, which generate new cartilage on the GP. The chondrocytes will be slowly replaced by osteogenic progenitor cells and osteoblasts to produce the bone.^84^ Interestingly, under normal conditions p53 acts as a negative regulator of osteoblastogenesis by repressing the promoter activity of transcription factors required in the early phase of osteogenic commitment, such as Osterix,^92^ Cbf*α*-1, and Runx2 in osteoprogenitor cells^73, 93^ (Figure 2). According to these evidences, p53-null mice are considered as a model for increased bone remodeling and osteosclerosis.^94^ During MSC osteogenic differentiation, p53 can inhibit osteogenesis^95, 96^ along with the downregulating expression of critical osteogenic transcription factors including Osterix and Runx2.^92^ Higher bone density and formation rate have been reported in p53-deficient mice along with increased bone resorption, which is not directly regulated by p53.^92^

### MSCs are a source of osteoblasts

MSCs are a subset of adult progenitor cells that exist in almost all adult tissues (bone marrow, adipose tissue, skin, and liver). Adult MSCs have proven to be cells of mesodermal origin, which can give rise to skeleton, muscle, heart, spleen, and other internal organs.^97^ These cells exert a key function in the maintenance of tissue homeostasis, tissue regeneration, and wound repair.^98^ MSCs show immunoregulatory properties,^99^ self-renewing, and differentiation ability into mesenchymal lineages (i.e. chondrocytes, osteoblasts, adipocytes, endothelial cells, pericytes).^100, 101^ P53 can regulate key transcription factor genes involved in exclusive programs of differentiation and dedifferentiation of somatic cells, with an impact on stemness and development. Given that p53 is a tumor suppressor gene and gate keeper of cell differentiation, in this review we want bring to light the connection of p53 status in MSCs, BME, and OS development. Notably, p53-null MSCs exhibit accelerated growth rate and aberrant osteogenic differentiation compared with wild-type MSCs, which contributes to tumor bone formation. Indeed, distinct studies highlight that p53-deficient bone marrow-derived MSCs can proliferate faster, and appear to differentiate earlier during *in vitro* osteogenic differentiation compared with the wild-type MSCs.^93, 95, 102^ However, this ‘tricky' appearance to differentiate earlier into osteoblasts reflects a more complex scenario; indeed, p53-null MSCs are impaired in achieving terminal differentiation towards mature osteocytes.^92^ MSCs represent a source of precursor for osteogenic progenitor cells and osteoblasts. P53 mutations that lead to defects in the control of cell growth of osteogenic progenitor cells represent the main source of sporadic OS.

The *in vitro* knockdown of p53 in mouse embryonic fibroblasts (MEFs), which are cells representative of an embryonic stage of development, induced higher expression level of Osterix and Runx2^73^ but not of terminal differentiation markers such as Osteocalcin and Sost-Sclerostin.^103^ Conversely, p53 knockdown in multipotent bone marrow stromal cells (*MBA*-15), which resembles adult progenitor cells,^73, 104^ promoted terminal osteogenic differentiation.^73^ Consistently, also after reintroduction of wild-type p53 in OS cell line, apoptosis and terminal differentiation were promoted.^104^ We can emphasize that p53 can regulate bone formation and the differentiation of early osteogenic precursors as demonstrated by the knockdown of p53 in MEF, and, furthermore, it can also promote terminal differentiation in *MBA*-15.

## P53 and MSCs

### P53 and MSC proliferation

**Figure 3:**
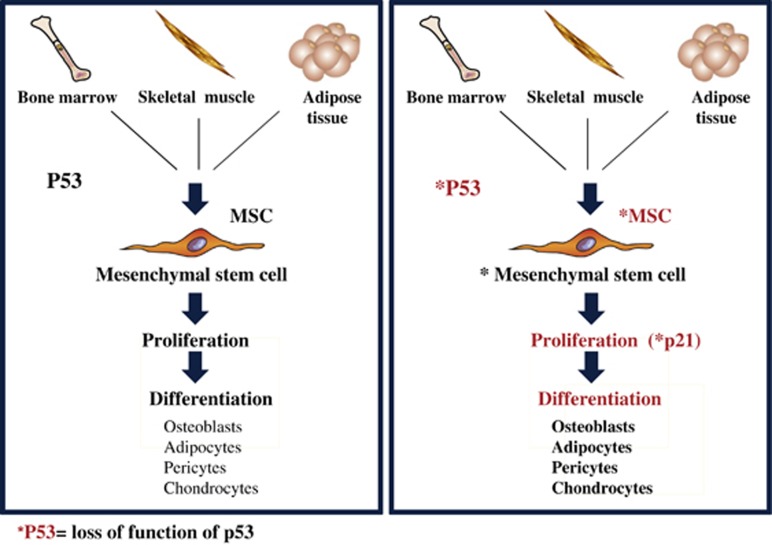
Loss of function of p53 compromises proliferation and differentiation of MSCs. MSCs can be isolated from adult organs, such as bone marrow, skeletal muscle, adipose tissue, and others, with a higher prevalence from the bone marrow (BM). MSCs can be identified *in vitro* for their surface markers and their multipotential differentiation properties. P53 has a role in regulating growth and proliferation of MSCs. Mutational events of p53 or p53 deficiency compromise the proliferation rate of MSCs mainly through p21 or CDK inhibitor p21^Cip1/Waf1^

Isolated MSCs can be maintained in culture *in vitro* for several passages without being severely compromised in their properties.^105^ The induction of p21 or cyclin-dependent kinase (CDK) inhibitor p21^Cip1/Waf1^ mainly promoted by p53 is associated with cell-cycle arrest (Figure 3). This suggests that alterations in cell-cycle regulators represent one of the main causes inducing aberrations in MSCs.^106^ Transformation of MSCs is highly correlated with simultaneous abnormalities of p53 and p21, and this could represent the event that could lead to the origin of mesodermal tumors.^106^ Nevertheless, it has been proven that loss of p53 in MSCs promote higher growth rate, chromosomal instability, resistance to apoptosis, and senescence^107, 108^ (Figure 3). Interestingly, p53 has a key role in regulating both differentiation of mesenchymal precursors and quiescence of hematopoietic stem cells in the bone marrow environment.^109^

Notably, bone marrow is one of the important sites for hematopoiesis in adults where hematopoietic stem cells are kept in a stemness condition.^100, 110^ Bone and bone marrow are functionally and anatomically correlated^87^ composing a unique compartment, which has a role in hematopoiesis and in bone homeostasis.^100, 111^ Transplanted nestin-marked human MSCs into the bone marrow cavity of nonobese diabetic/severe-combined immunodeficiency mice persisted 10 weeks after transplantation. Interestingly, these transplanted cells were able to differentiate into all the cells of the hematopoietic environment.^112^ Indeed, nestin-positive cells in the bone marrow have been recognized to have all the properties of MSCs, and were closely located with hematopoietic stem cells to support their quiescence.^113^ *In vivo* depletion of nestin-positive cells reduced the percentage of hematopoietic progenitor cells hosted in the bone marrow.^113^

### P53 and MSCs differentiation

**Figure 4:**
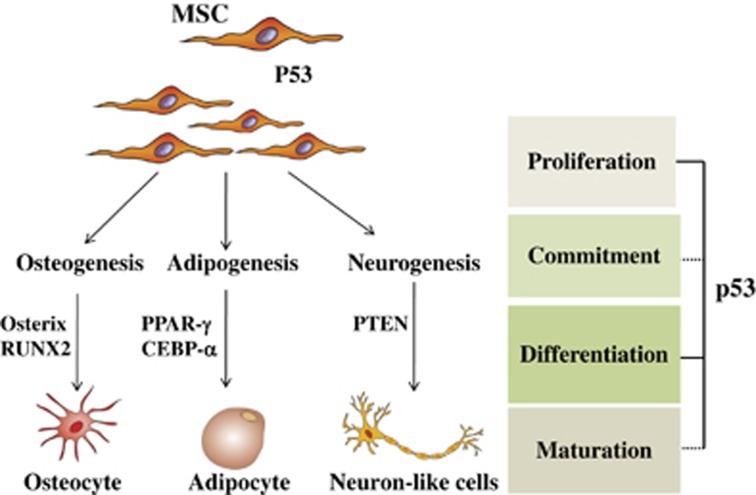
P53 is a negative regulator of differentiation pathways of MSCs. P53 can negatively regulate differentiation of mesenchymal progenitor cells such as osteogenesis, myogenesis, adipogenesis, and neurogenesis pathways by downregulating the expression levels of key transcription factor genes. In undifferentiated MSCs, p53 maintains lower expression levels of key transcription factor genes involved in the early phases of differentiation, such as Osterix and Runx2 for osteogenesis, PPAR-*γ*, CEBP-*α* for adipogenesis, Rb for myogenesis, and PTEN for neurogenesis. P53 status exerts a decisive role on proliferation, commitment, differentiation, and maturation of MSCs

*In vitro* gene knockdown of *TP53* and *Trp53* have revealed the importance of p53 in mesenchymal differentiation of multiprogenitor cells. However, controversial role of p53 during differentiation of MSCs have been reported.^92, 95^ Cell cycle and differentiation represent two interconnected processes in which p53 can exert distinct functions depending on the cell type. Absence of p53 can block the terminal cell differentiation, resulting in the accumulation of early and intermediate progenitors, which can lead to alterations of that specific differentiation pathway such as osteogenesis, adipogenesis, or myogenesis (Figure 4). For example, p53 negatively regulates adipogenesis by repressing the key adipogenic transcription factors PPAR-*γ* (proliferator-activated receptor-*γ*) and C/EBP-*α* (CCAAT/enhancer-binding protein-*α*).^74, 114^ Adipocytes and osteoblasts are both cells derived from multipotent progenitor cells. The undifferentiated status of the cells is kept by the repression of transcription factors that repress each other to preserve multipotency. However, upon appropriate stimulations, MSCs make sequential cell fate choices.^115^ The commitment of MSCs towards a specific mesenchymal differentiation program is coordinated also by p53, which regulates their multipotential state. In *in vivo* studies, p53 is upregulated in adipocytes from genetically obese mice in a fed state. Transgenic mice overexpressing functional p53 gain less body mass and adipose tissue when compared with wild-type mice; this has suggested an inhibitory role for p53, which may be exerted by changes in metabolism.^116^ Indeed, in the absence of functioning p53, a shift from oxidative phosphorylation towards glycolysis was observed.^117^ Furthermore, even myogenic differentiation can be monitored by p53. Distinct studies have shown an increase in *p53* mRNA levels during myogenic differentiation *in vitro*.^118, 119, 120^ P53 might be involved in this process by regulating the retinoblastoma protein, Rb, which has a pivotal role in the differentiation of muscle through cell-cycle arrest and also by specific genes involved in the myogenic differentiation program.^121, 122, 123^ The association of p53 expression and cell-cycle regulators, which are target genes of p53, such as p21, was observed during the development of the mouse nervous system.^124, 125^ Indeed, p53 monitors the differentiation of neural stem cells via its regulating pathways including cooperation with phosphatase and tensin homolog (PTEN).^125^ Notably, accumulation of mutant p53 in neural stem cells in the subventricular zone of the brain could generate aberrant neural progenitor cells and promote glioma formation.^126^ However, it is still unclear how p53 functions in the specific signaling context to regulate neural stem cells differentiation.^127^ In hematopoiesis, p53 induces hematopoietic stem cells to differentiate into proper mature blood cells and function in maintaining their quiescence.^73^ So far, the development of skeletal muscles and blood system has not yet been found abnormal in p53-null mice. Several *in vitro* studies have confirmed the role of p53 as a negative regulator in cell differentiation pathways, which reflects the complexity of the underlying mechanisms (Figure 4).

## P53 and MSCs dictating tumor microenvironment

### P53 and tumor microenvironment

Interestingly, our previous work has proved that MSCs that lack p53 exhibit tumor-promoting characteristic through high secretion of nitric oxide (NO) and higher vigorous immunomodulation when compared with wild-type MSCs.^128^ We found that the higher secretion of NO from p53-deficient MSCs have an inhibitory effect on T cells, and promote tumor growth.^128^ Thus, loss of p53 function in MSCs can promote their transformation by regulating their immunoproperties, growth, and proliferation. Importantly, p53 can dictate tumor microenvironment in an MSC-related manner. Indeed, except for tumor cells, also *non*-tumoral cells in tumor stroma were reported to gain *p53* mutations, which were associated with regional lymph-node metastases.^129^ When p53 is inactivated, tumor stroma shows less response to anticancer drugs, such as cisplatinum, etoposide, and vincristine as a consequence of failure in upregulating p53-inducible genes and inducing apoptosis of tumor cells.^130^ Alternatively, ablation of p53 in tumor stroma has promoted tumor growth by upregulating the expression of stromal cell-derived factor 1/C–X–C motif chemokine 12.^131, 132^ Notably, tumor stroma with dysfunctional p53 can enhance differentiation of myeloid-derived suppressor cell, exacerbate immunosuppression, and promote tumor progression.^133^ Furthermore, the role of p53 in the tumor microenvironment under hypoxic conditions has also been reported. Indeed, p53 targets the subunit HIF-1-*α* of hypoxia-inducible factor (HIF), necessary for metabolism adaptation, avoiding its proteasomic degradation through murine double minute 2 protein.^134^ Ablation of p53 increases the expression of HIF-1-*α* in a hypoxia condition, which in turn induces the expression of vascular endothelial growth factor (VEGF) in tumor cells and promotes angiogenesis, neovascularization, tumor growth, and invasion. VEGF and HIF-1-*α* are, furthermore, overexpressed in several types of human cancers, especially with HIF-1-*α* in metastatic OS.^135^

### P53 and MSC in OS tumor microenvironment

**Figure 5:**
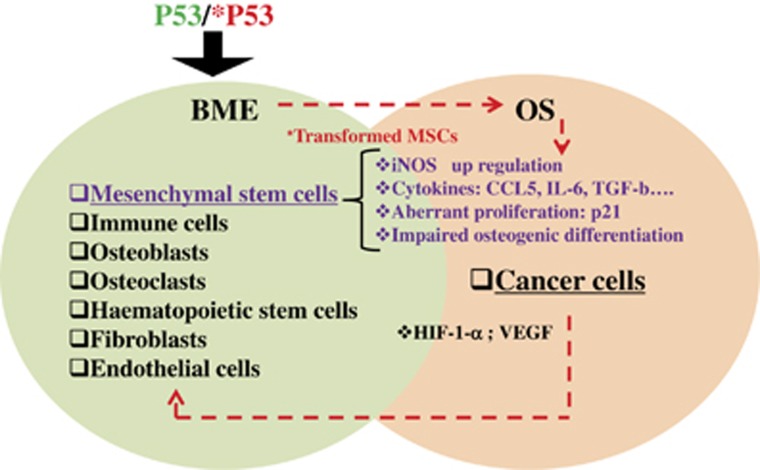
P53 status and the vicious cycle between BME and OS. In a p53 mutational landscape, BME components represent a soil for tumor promotion. In this scenario, p53-null MSCs exhibit higher proliferation rate, impaired osteogenesis, and altered immunoregulatory properties such as higher expression levels of iNOS (inducible nitric oxide synthase), CCL5, IL-6, and TGF-*β*. These molecules can, in turn, stimulate bone tumor-forming cells to produce cytokines to support their growth and affect BME. This generates a vicious cycle of cross-talking between BME and OS, which is dictated from p53 status

During bone growth, several factors and extracellular matrix components secreted by mesenchymal progenitor cells and chondrocytes in the GP will recruit BME components to guarantee a balanced bone remodeling. The BME hosts different types of cells, including osteoblasts, osteoclasts, mesenchymal cell precursors, hematopoietic stem cells, chondrocytes, and endothelial cells, as well as fibroblasts stromal cells and immune cells. In this special scenario, p53 status has a determinant role (Figure 5). In the context of bone remodeling, along with the coexistence of aberrant conditions arising from a p53 mutational landscape, the BME components could contribute to altered bone homeostasis compromising the cross-talk between MSCs, osteoblasts, osteoclasts, and hematopoietic cells, and therefore it dictates the environment for tumor initiation.^84^ Given the plasticity of MSCs to generate and differentiate into several cell types, including osteoblasts, it is not surprising that MSCs with p53 aberrations have been suggested to be the cells of origin for bone tumor, including OS, chondrosarcoma, Ewing's sarcoma, and sarcoma.^75^ We previously have discussed about the impaired osteogenic differentiation of p53-deficient MSCs; however, we here want to emphasize that p53-null MSCs also represent an important cellular component of tumor microenvironment. Interestingly, it has been demonstrated that in tumor BME, MSCs can support OS growth through the expression of CCL5 (chemokine ligand 5).^136^ Interestingly, CCL5/CCR5 (C–C chemokine receptor type 5) axis can promote OS migration through the extracellular signal-regulated kinase pathway, which induces the nuclear factor κ-light-chain enhancer of activated B cells (NF-κb).^136^ Conversely, growth factors secreted from cancer cells and during bone resorption, such as tumor necrosis factor-*α*, TGF-*β*, bone morphogenetic protein 2 (BMP2), and interleukin-6 (IL-6), can promote osteoclast maturation by directing the expression of RANK (receptor activator of the receptor activator of nuclear factor-κb) on the surface of osteoclast precursor cells increasing bone erosion. The increased bone degradation culminates in the release of BMP2 and TGF-*β*, which severely contribute to tumor growth and stimulate the osteoclast activity.^137^ TGF-*β* can evoke MSC-secreted IL-6, which acts by promoting OS metastasis via STAT-3 (signal transducer and activator of signal-3).^138^ These data emphasize how the loss of function of p53 is a determinant in dictating the conditions that contribute to initiate OS: on the one hand, it can affect proliferation, immunoproperties, and compromise osteogenesis of undifferentiated MSCs, but on the other hand, it can affect the properties of BME components compromising the talk between BME and cancer cells, a further condition that supports OS initiation and development.

## Conclusion

In this review, we aimed to bring to light that p53 has a pivotal role in keeping the balance between bone formation and bone degradation. Indeed, p53 not only regulates the genomic stability of MSCs but also their osteogenic differentiation functioning as ‘bone remodeling surveillant' to prevent bone tumor initiation. *Wang et al.*^92^ and other groups have proposed a negative role of p53 in regulating osteogenesis and other mesenchymal differentiation programs. We set the evidence that p53 mutational events occurring in undifferentiated MSCs or in osteoblasts at different stages of commitment can promote OS initiation^72^ as a consequence of alterations of osteogenic differentiation, bone remodeling, and bone homeostasis.^139^ Indeed, OS is a heterogeneous tumor that includes cells at different stages of commitment during osteogenesis.^140^ Interestingly, the osteosclerotic condition observed in p53-null mice imposes the phenotype of OS development.^106^ We summarize that p53 can affect osteogenic differentiation of MSCs and largely contribute to OS initiation: (1) it can promote or abrogate differentiation of multipotent progenitor cells acting as a negative mediator of transcription factors of early osteogenic differentiation; (2) it can regulate the genomic stability, growth, and proliferation of MSCs; (3) it can affect the immunoproperties of MSCs through growth factors and chemokine secretion; (4) it can affect the BME-regulating immune properties, growth, proliferation, and differentiation of microenvironment components. Further investigations on the molecular mechanisms through which loss of function of p53 can affect properties of MSCs and osteoprogenitor cells should be considered. This will ameliorate the knowledge of p53 function in the context of bone biology, and also will be helpful in identifying new strategies for targeting key molecules necessary for OS formation and survival.

## Abbreviations

OS: osteosarcoma
MSC: mesenchymal stem cells
BME: bone tumor microenvironment
Rb: retinoblastoma gene *RB1*; retinoblastoma protein
TGF-*β*: transforming growth factor-*β*
GP: growth plate
Cbf*α* -1: core-binding factor *α*-1
MEF: mouse embryonic fibroblast
*MBA*-15: multipotent bone marrow stromal cells
CDK: cyclin-dependent kinase
PPAR-*γ*: proliferator activated receptor-*γ*
C/EBP-*α*: CCAAT/ enhancer binding protein-*α*
PTEN: phosphatase and tensin homolog
NO: nitric oxide
MDSC: myeloid-derived suppressor cell
HIF: hypoxia-inducible factor
VEGF: vascular endothelial growth factor
CCL5: chemokine ligand 5
BMP2: bone morphogenetic protein 2
IL-6: interleukin-6
